# Inulin-Stabilised Vegetable Oil Emulsions as Fat Replacers in Chicken Frankfurters: Technological and Textural Evaluation

**DOI:** 10.3390/gels11110863

**Published:** 2025-10-28

**Authors:** Tamara Stamenić, Sladjana Šobajić, Maja Petričević, Nikola Delić, Slobodan Dolašević, Slaviša Stajić, Nikola Stanišić

**Affiliations:** 1 Institute for Animal Husbandry, Autoput za Zagreb 16, 11080 Belgrade, Serbia; tstamenic169@gmail.com (T.S.); majanovakovic@live.com (M.P.); delicnikola68@yahoo.com (N.D.); dolasevicslobodan.izs@gmail.com (S.D.); 2 Department of Bromatology, Faculty of Pharmacy, University of Belgrade, Vojvode Stepe 450, 11221 Belgrade, Serbia; sladjana.sobajic@pharmacy.bg.ac.rs; 3 Faculty of Agriculture, University of Belgrade, Nemanjina 6, 11080 Belgrade, Serbia; stajic@agrif.bg.ac.rs

**Keywords:** frankfurters, fat replacement, inulin-based emulsion gels, texture profile analysis (TPA), storage stability

## Abstract

This study investigated the complete replacement of pork backfat in frankfurters with inulin-based emulsion gels made from linseed, walnut or algal oil and structured in two ratios (1:2:0.5 and 1:2:1, oil–water–inulin). Proximate composition, water holding capacity, emulsion stability and colour were assessed after production, while texture profile analysis (TPA) was monitored during 45 days of vacuum storage. The reformulated sausages showed a significant reduction in fat content (from 21.91% to 3.81%, *p* < 0.001) and increased water and carbohydrate levels (*p* < 0.001). These shifts in composition resulted in a slightly lower pH, higher cooking and purge losses and lower emulsion stability (*p* < 0.001), particularly when treated with algal oil. Colour measurements revealed lighter (higher L*, *p* = 0.008) and more yellowish sausages (*p* < 0.001), with walnut oil at a 1:2:0.5 emulsion ratio showing the least deviation from the control (ΔE = 7.45). The TPA showed that oil type was the dominant factor. Walnut formulations, especially in the 1:2:1 ratio, had hardness and chewiness values closest to those of the control, while algal sausages were softer and less cohesive (*p* < 0.05). PCA and heatmap analyses confirmed clustering by oil type and storage time, underlining the technological suitability of the walnut gels. Overall, inulin–oil gels enable nutritional reformulation but pose a technological challenge, with walnut oil proving to be the most promising substitute and algal oil requiring additional stabilisation.

## 1. Introduction

Fat is a key component of emulsified meat products, such as frankfurters and Bologna-type sausages, and contributes to flavour, juiciness, mouthfeel and texture stability [1,2]. Traditionally, pork fat accounts for 20–30% of these formulations, but its high saturated fat and cholesterol content has been directly linked to cardiovascular disease, obesity and other chronic diseases [3,4,5]. In response, consumer demand for healthier meat products has led to reformulation strategies aimed at reducing or replacing animal fat while maintaining product quality [6,7].

One common approach is to replace pork fat with vegetable oils. However, the direct incorporation of liquid oils into meat matrices often leads to impaired texture, reduced juiciness, higher cooking loss and increased oxidative instability [1,8]. To address these limitations, gelled emulsion systems have gained attention as innovative fat substitutes, as they combine the structuring of oil with the immobilisation of water in a three-dimensional gel matrix [9]. By stabilising oil droplets in biopolymer networks, these gels can mimic the technological and sensory functions of animal fat. Recent advances in oleogels, nanocellulose-based gels and cold-set emulsions highlight their potential to improve water and fat binding, reduce cooking loss and increase oxidative stability in reformulated meat products [5,7,10,11].

Among the structuring agents, inulin has attracted particular attention due to its dual role as a fat substitute and fibre source [12]. Inulin gels have a creamy texture and high water holding capacity and can stabilise emulsions with vegetable oils [6,7,13]. From a gel science perspective, inulin can form thermoreversible networks through hydrogen bonding, whereby the concentration and dispersion conditions have a decisive influence on gel strength, water immobilisation and microstructural integrity. Studies have shown that inulin-based gels improve the fatty acid profile, reduce the energy density and increase the storage stability of low-fat meat products [14]. In particular, the oil–water–inulin ratio determines the rheological and technological performance of the gel: higher inulin contents generally improve emulsion stability and water binding, while lower concentrations lead to softer gels with weaker structural integrity [13]. Inulin addition has also been associated with favourable textural properties, such as improved elasticity and adhesiveness, in sausage formulations [15,16,17].

In addition to the structuring agent, the choice of oil is also of nutritional importance. Linseed and walnut oils are rich in α-linolenic acid (ALA), which reduces the SFA content and enriches the n-3 fatty acids in reformulated meat products [5,18]. Algal oil complements these plant sources by providing long-chain n-3 fatty acids such as eicosapentaenoic acid (EPA) and docosahexaenoic acid (DHA), which are important for cardiovascular and metabolic health [19,20,21]. Reformulation with these oils improves the PUFA/SFA ratio and optimises the n-6/n-3 balance, which is in line with current dietary recommendations to reduce SFA intake to below 10% of daily energy while increasing unsaturated fatty acids [22]. When structured in inulin gels, these oils not only provide nutrient fortification, but also benefit from the gel’s protective capacity against oxidation and phase separation, providing a synergistic approach to product quality [14,23].

Colour and emulsion stability are other critical quality characteristics of reformulated sausages. The colour parameters (L*, a*, b*) have a significant impact on consumer acceptance, while emulsion stability reflects the ability of the gel to bind water and fat during thermal processing and storage. Emulsions stabilised with inulin have been reported to minimise liquid separation, reduce cooking loss and improve colour uniformity compared to direct oil addition [13,24]. These gel properties are particularly important for frankfurter-type sausages, where microstructural stability has a direct impact on sensory quality.

Despite these promising developments, research on inulin-stabilised emulsions in frankfurter-type products is still limited. Most studies focus on improvements in composition or nutritional value, while systematic evaluations of the gel’s functionality, particularly its role in stabilising emulsions, modulating colour and maintaining texture during storage, are scarce [6,9,25,26,27].

This study builds upon our previous work on frankfurters reformulated with oil–inulin emulsions [28], which demonstrated significant improvements in the lipid profile, including a reduction in total saturated fatty acids and cholesterol by up to 30%, as well as enrichment with ALA, EPA and DHA. However, oxidative stability was impaired during cold storage, especially for linseed and algal oil. Sensory evaluation revealed that walnut oil had the most acceptable flavour and aroma, while algal oil caused off-flavours. However, the combined effect of oil type, emulsion ratio, and storage stability in a fully formulated product such as chicken frankfurter has not yet been systematically examined. The present study addresses this gap by evaluating the influence of three oil types (linseed, walnut, and algal oil), two emulsion ratios, and refrigerated storage (45-day vacuum storage) on the technological, colour, and texture characteristics of inulin-based reformulated frankfurters. By linking the gel composition with the technological and textural results, this study provides new insights into the function of inulin-based gels as a structuring agent in the reformulation of healthier frankfurter-type sausages.

## 2. Results and Discussion

### 2.1. Proximate Composition

The proximate composition showed clear differences between the control and the reformulated sausages (Table 1). Replacing the backfat with inulin–oil gels resulted in a significant reduction in fat content (*p* < 0.001), from 21.91% in the control to approximately 5% in the reformulated treatments. This reduction was accompanied by a proportional increase in carbohydrate content, reflecting the contribution of inulin to the formulation (*p* < 0.001). These results confirm previous studies that have shown that substitution with inulin effectively reduces the fat content while enriching the carbohydrate content, thereby increasing the fibre content of meat products [7,14].

The protein content decreased slightly but significantly in the reformulated samples (*p* = 0.012), probably due to dilution by the addition of water and inulin. Similar effects were observed in other fat-reduced meat products produced with biopolymer gels [1]. The water content, on the other hand, increased significantly in all reformulated groups (*p* < 0.001), which is consistent with the strong water-binding capacity of inulin gels compared to pork backfat [24]. The ash content was not significantly affected by the treatment (*p* > 0.05).

Among the oils, all reformulated treatments exhibited comparable proximate compositions, with only minor variations between linseed, walnut, and algal oil gels. These results demonstrate that the primary compositional changes are attributable to the fat replacement process itself rather than the specific oil source.

### 2.2. Physicochemical Properties

The pH values of the frankfurters ranged from 6.11 to 6.22, with the control group showing significantly higher values (*p* < 0.001) compared to the inulin-based reformulations (Table 1). This slight reduction is consistent with previous studies, which report that the incorporation of inulin or the substitution of vegetable oil tends to lower the pH, likely due to differences in ionic composition, protein solubility, and buffering capacity relative to pork fat [17,29]. Comparable trends have also been described in chia- based gel systems [1].

In contrast, water-holding capacity (WHC) was not significantly affected by treatment (*p* > 0.05). All formulations showed values close to 1.0, indicating that the complete replacement of pork backfat with inulin–oil gels does not affect water retention. This stability can be attributed to the hydrophilic nature of inulin, which improves water binding through hydrogen bonding and immobilisation within the gel matrix [24].

Cooking loss increased significantly (*p* < 0.05) in the reformulated sausages, particularly in the walnut and algal oil treatments, with the highest values recorded in the 1:2:0.5 formulations (Table 1). This result is consistent with previous studies that have shown that partial or total fat replacement with inulin–oil gels can weaken the cohesion of the matrix during heating, resulting in greater fluid release [14]. Similarly, purge loss during refrigerated storage was also significantly higher for the reformulated samples (*p* = 0.043), suggesting that the modified fat-gel network retains liquids less effectively under vacuum conditions. Nevertheless, the magnitude of this effect remained limited, confirming that the overall water-binding ability of the reformulated systems was acceptable.

### 2.3. Emulsion Stability

The greatest treatment differences were observed in the emulsion stability parameters (WL, FL and TFL), as shown in Table 1. Water loss was significantly higher in the reformulated sausages, with linseed and walnut oil gels showing the highest exudation, followed by algal oil, while the control group showed the lowest values (*p* < 0.001). Fat loss followed a similar pattern and was increased in all reformulated samples, with the algal oil gel at 1:2:0.5 emulsion ratio showing the lowest stability (8.15%). This observation is consistent with reports that marine oil emulsions are more susceptible to destabilisation upon heating if they are not adequately structured [1,30,31,32]. Consequently, the TFL was significantly higher in the reformulated groups (*p* < 0.001), confirming that the pork backfat provides better emulsion stability than the inulin–oil gels.

From Table 1 it is also shown that emulsion stability of vegetable oils with 1:2:1 gels is generally performing better than 1:2:0.5 formulations. This supports the view that a higher inulin content strengthens the gel network and improves the immobilisation of oil droplets and water molecules [33].

Overall, these results emphasise the multifactorial effects of replacing pork back fat with inulin–oil gels. The shift in proximate composition, particularly the significant reduction in fat content and corresponding increase in water and carbohydrate content, resulted in a fundamentally different product matrix that directly affected functional properties. Slightly lower pH values and an unchanged WHC indicate that the protein–water interactions remained relatively stable despite the fat replacement. However, the weaker structural cohesion of the gel-oil matrix compared to pork fat was reflected in higher cooking and purge losses and lower emulsion stability, especially for highly unsaturated oils such as linseed oil and algal oil. Increasing the inulin content (1:2:1) partially mitigated fat separation, highlighting the role of inulin concentration in strengthening the gel network, but it was not sufficient to fully offset the destabilising effects of oil type. Taken together, these results show that although inulin–oil gels enable a nutritional reformulation of frankfurters, their technological performance is still highly dependent on the interplay between composition and processing stability.

### 2.4. Colour Properties

Colour is a critical sensory and technological characteristic of sausages that influences consumer acceptance and perceived freshness. The replacement of pork back fat with inulin-based emulsion gels significantly influenced all colour parameters (Table 2).

Lightness (L*) increased in all reformulated treatments compared to the control, with the algal oil samples achieving the highest values (*p* = 0.008). A similar increase in L* was reported by de Souza Paglarini et al. [14] in Bologna-type sausages reformulated with inulin gels, and by Franco et al. [7] in frankfurters containing linseed oleogels. This effect is often attributed to a higher moisture content, which dilutes the pigment concentration and increases light scattering in the meat matrix. Emulsion ratio also affected L*, with the 1:2:1 formulations yielding lighter products than 1:2:0.5, for walnut and algal group, suggesting a synergistic role of oil colour and gel structuring in altering surface reflectance.

Redness (a*) decreased significantly in all reformulated sausages, with algal oil at a 1:2:1 emulsion ratio showing the lowest values (*p* < 0.001). This decline in redness likely results from the combined effects of pigment dilution and enhanced lipid oxidation associated with the high unsaturation of vegetable oils, leading to partial discolouration of myoglobin [34].

The values for yellowness (b*) and hue angle (h*) increased significantly in all reformulated groups, with the greatest effect observed in the algal oil treatment at a 1:2:1 emulsion ratio (*p* < 0.001). Similar results were reported in previous studies by Câmara & Pollonio [35] and Franco et al. [7], who showed that replacing pork backfat with linseed oil or linseed oil oleogels resulted in a linear increase in L* and b* values, while Stajić et al. [36] also found higher yellow colouration in fermented sausages enriched with linseed oil preparations compared to controls. These shifts can be attributed to the intrinsic pigment characteristics of the added oils, golden tones in linseed oil and green-yellow hues in algal oil, as well as their uniform distribution within the inulin gel matrix, which improves light scattering and accentuates the yellow/hue components.

Chroma (C*), which represents colour saturation, also varied depending on oil type and inulin content (*p* < 0.001). Algal and linseed oil at a 1:2:1 emulsion ratio produced the most saturated colour tones, indicating more vivid and intense colour tones compared with the control. In contrast, both walnut oil groups (at 1:2:0.5 and 1:2:1 emulsion ratios) did not differ statistically from the control.

Total colour difference (ΔE) confirmed perceptible deviations (ΔE > 3) between the control and the reformulated samples. The smallest ΔE was observed for walnut oil formulations, suggesting a closer resemblance to the control, while the algal oil treatments, particularly 1:2:1, exhibited the highest colour deviation (ΔE = 17.03), indicating a more distinct appearance.

Overall, the colour shifts can be directly linked to the composition and stability results described in Table 1: Higher water content and lower fat content contributed to greater lightness, while weaker emulsion stability promoted greater pigment dilution. In addition, oil-specific chromatic effects further influenced yellowness, hue and saturation. Walnut oil thus proves to be the most promising option for balancing nutritional improvements with visual acceptability, while algal oil remains a technological and sensory challenge despite its high nutritional value.

### 2.5. Texture Profile Analyses (TPA)

#### 2.5.1. Effect of Oil Type

Texture is an important quality attribute in emulsified meat products that strongly influences consumer acceptance through bite, juiciness and mouthfeel. In this study, the type of oil significantly influenced several TPA parameters (Table 3). Hardness was highest in the control and walnut formulations and lowest in the algal oil sausages, especially at a 1:2:1 emulsion ratio (*p* < 0.001). The firmer texture of the walnut samples can be explained by the relatively higher oxidative stability of walnut oil and the balanced PUFA profile, which appears to support a more coherent protein–gel network structure. This result is consistent with studies showing that reformulation with walnut oil emulsions maintains texture while improving lipid profiles in similar meat systems [31]. In contrast, algal oil, which is rich in long-chain PUFA and inherently more fluid, tends to disrupt emulsion stability (also reflected in the higher fluid loss in Table 1), resulting in weaker structural integrity and a softer texture, which is a limitation highlighted in broader studies on liquid vegetable oil incorporation without adequate gel structuring [9]. Similarly, Barbut et al. [2] reported that gels made with vegetable oils can either strengthen or weaken the sausage matrix, depending on the stability and structuring capacity of the oil. Cîrstea et al. [31] found that emulsion gels increased the firmness in Bologna sausages. They attributed this to the larger interfacial area of smaller vegetable oil droplets coated with proteins that increase resistance to compression. Youssef and Barbut [33] explained this effect as a result of denser protein–oil interactions. Furthermore, sausages with walnut oil in a 1:2:1 ratio had a firmer texture than samples with algal oil in a 1:2:1 ratio, suggesting that inulin concentration alone cannot compensate for the destabilisation caused by highly unsaturated oils. Delgado-Pando et al. [37] reported similar results with soy protein emulsions, where an improvement in texture was only achieved when oil type and gel structuring were compatible. The observed differences in hardness also correlate with emulsion stability and cooking loss data (Table 1). Sausages with higher emulsion stability and lower cooking loss, such as the walnut 1:2:1 formulations, exhibited firmer textures, whereas samples with weaker emulsion stability and greater fluid loss, such as the algal oil sausages, showed softer textures.

Adhesiveness also differed significantly among oils (*p* < 0.001), being higher (more negative) in control and walnut 1:2:1 emulsion ratio samples, indicating greater stickiness during compression. A lower adhesion force is technologically advantageous as it improves cutting, packaging and handling [38]. Cohesiveness and resilience were slightly reduced in walnut and linseed samples (at both 1:2:0.5 and 1:2:1 emulsion ratios), which is consistent with observations that certain oils can interfere with protein–inulin cross-linking [25]. Gumminess and chewiness were also reduced in the linseed and algal sausages compared to the control, while the walnut sausages had values closer to those of the full-fat product. Maintaining chewiness is particularly important for consumer perception of sausages, and these results highlight walnut oil as the most promising candidate among the oils tested.

In the present study, the emulsion ratio had little effect on springiness, suggesting that the inulin content affects strength but does not fully replicate the elastic properties of pork backfat. This finding is consistent with the results of Pintado et al. [1], who also observed an increase in hardness and chewiness with gelled oil emulsions, but found no significant improvements in springiness.

Overall, the type of oil had a marked impact on mechanical resistance and surface properties, with walnut oil producing a texture most comparable to the control and algal oil yielding the softest, least cohesive structure.

#### 2.5.2. Effect of Storage

The storage time significantly increased the hardness, cohesiveness, gumminess, chewiness and resilience (*p* < 0.05), while the springiness and adhesiveness remained largely unaffected (Table 3). The control and walnut sausages showed a clear increase in hardness and gumminess, while linseed treatments exhibited minor or non-significant changes. The stiffening effect probably reflects the progressive protein cross-linking and water redistribution during refrigeration, a phenomenon commonly reported in emulsified meat [39]. Stajić et al. [38] similarly observed increasing hardness and chewiness during chilled storage of reformulated frankfurters, while Pintado et al. [1] reported a comparable increase in hardness and chewiness in oil gel-based frankfurters. Adhesiveness and springiness, however, showed no significant variation with storage time, supporting the stability of these parameters.

These results suggest that protein network tightening occurred mainly in the control and walnut groups, whereas the inulin-based emulsions with linseed or algal oil limited excessive hardening during storage. Despite these textural changes, cohesiveness and resilience remained within acceptable limits across 45 days, indicating that the inulin gels effectively stabilised the protein network and prevented excessive hardening or crumbling during storage.

#### 2.5.3. Interaction Effects

**Figure 1:**
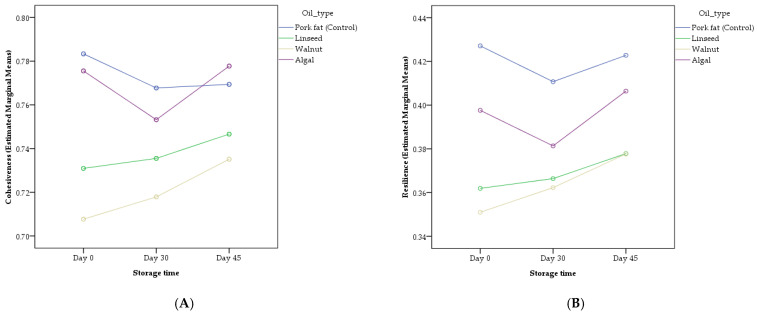
Interaction effects of oil type and storage time on the texture properties of chicken frankfurters reformulated with inulin-based emulsion gels. (**A**) Cohesiveness. (**B**) Resilience. Lines represent estimated marginal means derived from a two-way ANOVA model (Oil type × Storage time).

Significant interaction effects between oil type and storage time (O × S) were detected for cohesiveness (*p* = 0.011) and resilience (*p* = 0.014), whereas other texture parameters were unaffected (Table 3). As shown in Figure 1A,B, the effect of storage time on texture differed among oil types. Cohesiveness in the walnut and control sausages remained statistically stable throughout storage, whereas algal and linseed samples showed a slight but significant increase by day 45. Similarly, resilience increased significantly during storage for algal formulations, but not for linseed or control samples (same letters), suggesting that the more unsaturated oils underwent progressive structural rearrangement during chilled storage. Figure 1A,B demonstrate that the combined influence of oil composition and storage duration affected network elasticity more than either factor alone.

These trends may be associated with oil oxidation and emulsion destabilisation pro-cesses that occur during chilled storage, which affect the integrity of the protein–polysaccharide network differently depending on the fatty acid composition of the oil. Similar patterns were reported by Illippangama et al. [25], who observed that emulsions containing highly unsaturated oils undergo greater structural rearrangements during storage due to oxidative and moisture-related effects.

Taken together, these findings indicate that oil type remains the primary determinant of texture in inulin-based emulsion gels, while storage time mainly induces gradual structural tightening. The enhanced consistency of walnut-based formulations highlights their superior gel stability and oxidative resistance, supporting their potential for producing nutritionally improved chicken frankfurters with stable and acceptable texture throughout storage.

#### 2.5.4. Multivariate Relationships (PCA and Heatmap)

**Figure 2:**
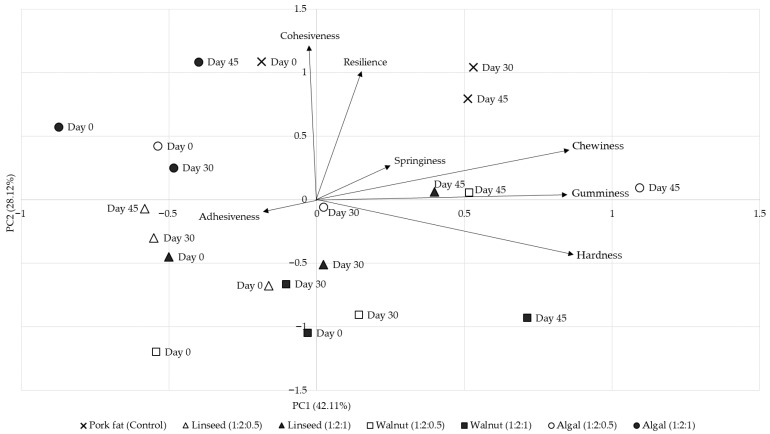
Principal component analysis (PCA) biplot of TPA parameters in chicken frankfurters reformulated with inulin-based emulsion gels. The biplot shows distribution of treatments and storage times relative to principal components PC1 (42.11%) and PC2 (28.12%). Vectors indicate loadings of individual texture parameters (hardness, cohesiveness, springiness, gumminess, chewiness, adhesiveness, resilience).

The PCA biplot (Figure 2) explained 70.23% of the total variance, with PC1 (42.11%) determined by hardness, gumminess and chewiness, while PC2 (28.12%) captured cohesiveness and resilience. This separation illustrates the trade-offs between fat substitution and gel structuring and demonstrates that firmness-related parameters dominate the variability in texture, whereas structural elasticity is captured on the secondary axis. The control sausages clustered separately from all reformulated samples, confirming the technological importance of pork back fat in defining traditional frankfurter texture. Among the reformulations, the walnut oil came closest to the control in a 1:2:1 ratio, suggesting that this formulation achieved the best balance between firmness and cohesiveness. In contrast, the algal oil treatments were furthest away, reflecting their weaker emulsion stability (Table 1), greater colour variation (Table 2) and softer, less resilient textures (Table 3).

**Figure 3:**
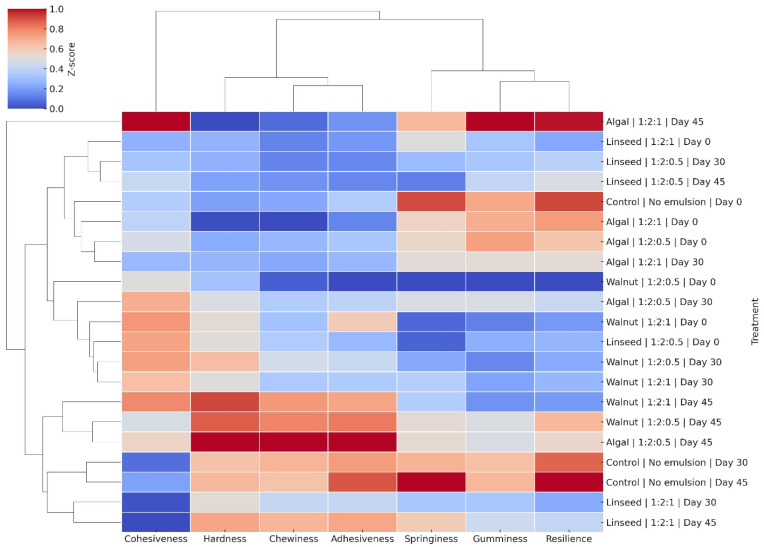
Clustered heatmap of texture profile analysis (TPA) parameters (hardness, cohesiveness, springiness, gumminess, chewiness, adhesiveness and resilience) in control and reformulated chicken frankfurters (linseed, walnut and algal oil emulsions; ratios 1:2:0.5 and 1:2:1) over 45 days of vacuum storage. The red shading indicates higher relative values, while the blue shading indicates lower relative values. Clustering was based on Euclidean distance and Ward’s linkage method.

The clustered heatmap (Figure 3) provided complementary insights by grouping the treatments and texture parameters simultaneously. Storage resulted in a progressive increase in hardness and chewiness (red shading), particularly in the walnut oil sausages, confirming the stiffening trends observed in Table 3. Conversely, the algal oil formulations consistently showed lower cohesiveness and resilience (blue shading), indicating a weaker gel matrix and a higher susceptibility to destabilisation. Within the oil types, the emulsion ratio influenced clustering: 1:2:1 formulations grouped closer to the control than 1:2:0.5, emphasising the role of higher inulin levels in improving network stability. This effect was most evident in walnut oil sausages, where the 1:2:1 gels not only improved emulsion stability but also maintained resilience during storage. In contrast, the type of oil remained the dominant destabilising factor in sausages made from algal oil, while the emulsion ratio had a weaker influence. Taken together, our PCA and heatmap analyses confirm that oil type is the most important factor for texture quality, while emulsion ratio has a secondary stabilising effect, which is particularly evident in walnut and linseed oil formulations.

### 2.6. Integrated Interpretation and Industry Perspective

The TPA results aligned closely with the proximate composition, emulsion stability and colour data, confirming the interdependence of compositional and functional quality attributes in reformulated sausages. Linseed and walnut gels, which exhibited higher emulsion stability, produced firmer textures, while algal oil sausages, which were characterised by greater fluid losses (Table 1), developed weaker and less elastic structures (Table 3, Figure 1, Figure 2 and Figure 3). The higher water content in the reformulated samples explained their lighter appearance (higher L*, Table 2) and slightly softer textures, while the lower redness (a*) could negatively influence consumer perception if not compensated by adequate chewiness and firmness. Multivariate analyses (PCA and heatmap) reinforced these trends and showed a clear clustering by oil type, with walnut—particularly in the 1:2:1 ratio—coming closest to the control group. This suggests that walnut oil emulsions achieve the most favourable balance between nutritional reformulation and technological functionality.

From an industrial perspective, lower adhesiveness and stable cohesiveness in reformulated sausages are favourable for slicing, packaging and high-throughput processing [1,40]. However, the lower resilience of algal oil formulations highlights the need for additional stabilisers or co-gelling agents to achieve a marketable texture. Choi et al. (2013) [4] and Domínguez et al. [5] emphasised that while oil gels offer clear nutritional benefits, technological trade-offs are unavoidable in large-scale applications. Recent work also shows that hybrid gels combining inulin with proteins, fibres or nanocellulose can improve both texture and oxidative stability [41,42], offering a promising route for future reformulation strategies.

## 3. Conclusions

This study showed that the complete replacement of pork back fat with inulin-based emulsion gels made from linseed, walnut or algal oil significantly changed the nutritional and technological profile of frankfurters. The reformulated sausages had a significantly lower fat content and a higher carbohydrate and water content, confirming the nutritional potential of inulin–oil gels. However, these shifts in composition reduced emulsion stability, increased cooking and purge losses and led to significant colour changes, in particular a lower redness and higher yellowness.

In terms of texture, the walnut oil gels, particularly in the 1:2:1 ratio, provided the most favourable firmness and chewiness and were closest to the control in the PCA and heatmap analysis. In contrast, the algal oil formulations were less elastic and cohesive, reflecting the destabilising influence of the long-chain PUFA. Nevertheless, the lower adhesiveness and stable springiness especially in walnut sausages are favourable for slicing and packaging efficiency.

From an industrial point of view, walnut and linseed gels seem to be the most promising for large-scale application, while algal systems require further stabilisation, for example, by co-gelling agents or reinforcement by antioxidants. Future research should investigate partial replacement strategies, consumer sensory validation and oxidative stability, in addition to the cost and scalability of gel preparation, to ensure that nutritional benefits are balanced with technological feasibility and market acceptance.

## 4. Materials and Methods

### 4.1. Materials

Smoke-permeable collagen casings (22 mm in diameter) were obtained from Koteks Viscofan (Novi Sad, Serbia). A standardised spice mix and functional ingredient blend were supplied by Fenc Company (Novi Sad, Serbia). Walnut and linseed oils (cold-pressed) were sourced from Linum doo (Čonoplja, Serbia), while algal oil (Omegatex^®^ ALGAE1060TG) was provided by Solutex (Madrid, Spain). Inulin (Fibruline™, Cosucra, Warcoing, Belgium) served as the main structuring component in emulsion preparation. All chemicals used for analytical procedures were of analytical grade.

### 4.2. Sausage Preparation and Experimental Design

All chicken frankfurters were produced at the pilot meat processing facility of the Institute for Animal Husbandry (Belgrade, Serbia). The control batch (C) was formulated using chicken breast meat, pork backfat, and ice water in a 50:25:25 (*w*/*w*) ratio. In the reformulated treatments, pork backfat was completely replaced with inulin-based emulsion gels containing linseed (L), walnut (W), or algal (A) oil as lipid sources. The selected inulin-to-water-to-oil ratios (1:2:0.5 and 1:2:1, *w*/*w*/*w*) were based on our earlier optimisation study [28] and further refined through preliminary screening of several ratios (1:2:0.25–1:2:1). The 1:2:0.25 formulations showed weak gel strength and excessive syneresis, while the 1:2:1 ratio yielded strong, cohesive gels with high oil retention and structural integrity. However, its high viscosity suggested that a less dense system could offer improved processability. Therefore, the intermediate 1:2:0.5 ratio was included in the present study to represent a more flexible, lower-structure emulsion while maintaining acceptable gel strength. Therefore, two gel systems were tested, differing in their oil–water–inulin ratios of 1:2:0.5 and 1:2:1 (*w*/*w*/*w*). They were selected to compare the effects of lower and higher gel structuring capacity, respectively, as inulin concentration strongly influences network strength, water immobilisation and texture [13,14,24].

To prepare the gels, inulin powder was dispersed in chilled distilled water (4 °C), hydrated for 30 min, and homogenised for 3 min at 6000 rpm using an Ultra-Turrax T25 homogeniser (IKA, Staufen im Breisgau, Germany). Oil was then added dropwise while the homogenisation speed was increased to 10,000 rpm, followed by an additional 3 min of homogenisation until a uniform gel was obtained. The prepared gels were stored at 4 °C for 12 h to ensure complete hydration, stabilisation of the network and uniform gelation before being incorporated into the meat batters.

Each formulation (C, L, W, A) was produced in triplicate as independent processing batches. For each batch, approximately 5 kg of batter was prepared, comprising chicken meat, the designated fat source (pork backfat or inulin-based emulsion gel), and ice/water. The basic formulation included the following additives (%, *w*/*w*): sodium chloride (1.8), nitrite curing salt (0.015), sodium tripolyphosphate (0.4), sodium erythorbate (0.04), soy protein isolate (2.0), sucrose (0.5), coriander (0.2), black pepper (0.15), and garlic powder (0.10). The production order of the treatments was randomised to avoid systematic processing bias.

Mixing was performed using a bowl cutter (Seydelmann K20, Stuttgart, Germany) at low speed (~1500 rpm) for 2 min followed by high speed (~3000 rpm) for 5 min, keeping the batter temperature below 12 °C. The homogenised mixture was stuffed into 22 mm collagen casings and thermally processed in a smoke chamber (Fessmann, Winnenden, Germany) according to the following schedule: drying at 50 °C for 10 min, smoking at 60 °C for 30 min, and cooking at 85 °C until the internal temperature reached 72 °C. Afterwards, sausages were cooled under running water, equilibrated for 24 h at 4 °C, vacuum-packed in low-oxygen-permeable polyethylene bags (OTR ≈ 20 cm^3^/m^2^/day at 23 °C), and stored under refrigeration (4 ± 2 °C).

The analyses were carried out at three points in time: (i) in the raw dough before stuffing, (ii) immediately after production (day 0) and (iii) after 30 and 45 days of vacuum storage. At each time point, nine sausages per treatment were randomly selected and analysed in triplicate.

### 4.3. pH, Water-Holding Capacity, and Processing Losses

The pH of the samples was measured immediately after preparation using a Hanna HI 83141 pH metre (Hanna Instruments, Smithfield, RI, USA) equipped with a puncture electrode. The instrument was calibrated with standard phosphate buffer solutions prior to use.

The water-holding capacity (WHC) was determined according to Zhuang et al. [43]. In brief, 10 g of uncooked batter was homogenised with 15 mL of 0.6 M NaCl solution in a centrifuge tube, cooled at 4 °C for 15 min and centrifuged at 3000× *g* for 15 min. The WHC (%) was calculated according to Equation (1):

(1) $$WHC\;(\%)=\frac{W1-W2}{W2}\;\times 100$$

where W1 refers to the weight of the solid material after centrifugation and W2 is the weight of the initial sample.

The cooking loss was calculated from the difference between the weights of the sausages after stuffing and after thermal processing and expressed as a percentage of the raw weight. Purge loss during storage was determined according to Pintado et al. [1].

### 4.4. Emulsion Stability (Water and Fat Binding Capacity)

Emulsion stability was evaluated as described by Bolger et al. [44]. Approximately 24 g of raw batter was placed in conical centrifuge tubes, heated to 98 °C in a water bath for 45 min, cooled in ice water for 10 min and inverted for 1 h to collect the released fluids on pre-weighed porcelain dishes. Total fluid loss (TFL), water loss (WL) and fat loss (FL) were calculated as follows:

- TFL (%) = (weight of drained fluid/initial weight of sample) × 100.
- WL (%) = (weight before drying − weight after drying)/initial weight of sample × 100.
- FL (%) = TFL − WL.

Lower fluid loss was interpreted as higher emulsion stability.

### 4.5. Proximate Composition

Proximate composition was determined on samples taken on day 0 (after production). Moisture content was quantified gravimetrically by oven-drying the samples at 103 ± 2 °C according to ISO 1442:2023 [45]. The protein content was determined using the Kjeldahl method, whereby the nitrogen content was converted to crude protein by a factor of 6.25, as described in ISO 937:2023 [46]. The lipid content was determined by Soxhlet extraction according to ISO 1443:1973 [47], while the mineral content (ash) was determined by incineration of the samples at 550 ± 25 °C according to ISO 936:1998 [48]. The carbohydrate content was calculated by difference.

### 4.6. Color Measurement

The instrumental colour was measured on the cut surface of the sausages immediately after production (day 0) using a Minolta CR-300 colourimeter (Minolta Camera Co., Ltd., Osaka, Japan), which had been previously calibrated against a standard white plate. The measurements were carried out under D65 illuminant conditions, with an observer angle of 10° and an aperture size of 8 mm. The CIE values L* (lightness), a* (redness) and b* (yellowness) were recorded. Chroma (C*) and hue angle (h*) were calculated using dedicated software. These assessments were carried out at room temperature (20 ± 2 °C). Three measurements were taken at different positions on each cross-section, and the mean value was calculated for each.

The total colour difference (ΔE) between the treatments was estimated according to Equation (2):

(2) $$\Delta E=\sqrt{{\left(L-L0\right)}^{2}+\;{\left(a-a0\right)}^{2}+\;{\left(b-b0\right)}^{2}}$$

where L0, a0 and b0 are the values of the control samples (C group).

### 4.7. Texture Profile Analysis (TPA)

The texture of the sausages was evaluated after production (day 0) and after 30 and 45 days of vacuum storage, using cylindrical samples (14 mm height × 16 mm diameter) taken from the centre part of each sausage. This was done following the procedure of Stajić et al. [38] with slight modifications. A TA.XT Plus texture analyser (Stable Micro Systems, Godalming, UK) equipped with a 25 mm aluminium compression plate (P/25) and a 5 kg load cell was used. Each sample was subjected to a double compression cycle up to 50% of its original height, with a pre-test speed of 60 mm/min, a test speed of 60 mm/min and a post-test speed of 300 mm/min. The following parameters were calculated from the resulting force-time curves: hardness, adhesiveness, springiness, cohesiveness, gumminess, chewiness, and resilience.

### 4.8. Statistical Analysis

Statistical analyses were conducted using SPSS version 22.0 (IBM Corp., Armonk, NY, USA). Before performing parametric tests, the data were assessed for normality using the Shapiro–Wilk test and for equality of variances using Levene’s test. A one-way ANOVA was used to evaluate the effects of treatment on proximate composition, physicochemical parameters, emulsion stability, cooking loss, purge loss, and colour attributes (Table 1 and Table 2). The factor “treatment” included the control (pork backfat) and six reformulated samples prepared with linseed, walnut, or algal oil at two emulsion ratios (1:2:0.5 and 1:2:1). When significant differences were detected (*p* < 0.05), means were separated using Tukey’s HSD post hoc test. For the texture profile analysis (TPA) data, a two-way ANOVA was conducted with Oil type (Control, Linseed, Walnut, Algal) and Storage time (0, 30, and 45 days) as fixed factors. Interaction effects (Oil × Storage) were also evaluated. When an interaction effect was found to be significant (*p* < 0.05), interaction plots were generated from estimated marginal means to visualise the combined effects of oil type and storage time on texture properties. All results are presented as mean ± standard deviation, and statistical significance was accepted at *p* < 0.05. Principal component analysis (PCA) was performed to analyse the multivariate relationships between the TPA parameters during storage. Sampling adequacy was checked with a Kaiser-Meyer-Olkin (KMO) index of 0.78 and Bartlett’s test of sphericity (*p* < 0.001), which confirmed sufficient intercorrelations. PCA was performed in SPSS and the biplots were exported and formatted in Microsoft Excel for clarity. A clustered heatmap was created in R (v4.3) using the pheatmap package. The data was standardised as z-scores over the texture parameters and hierarchical clustering (Euclidean distance, Ward method) was applied to both the rows (treatments) and columns (parameters). A diverging blue-white-red palette was used, with red representing above-average values and blue representing below-average values. This approach enabled the simultaneous visualisation of treatment clusters and covariations between texture attributes.

## Figures and Tables

**Table 1:** Effect of oil type and emulsion ratio on proximate composition, pH, and technological properties (WHC, cooking and purge losses, emulsion stability) of chicken frankfurters reformulated with inulin-based emulsion gels *.

	Control (C)	Linseed (L)		Walnut (W)		Algal (A)		*p* Value
		1:2:0.5	1:2:1	1:2:0.5	1:2:1	1:2:0.5	1:2:1	
Water (%)	59.68 ± 0.45 ^a^	72.44 ± 0.62 ^b^	71.03 ± 0.27 ^b^	72.58 ± 0.66 ^b^	71.21 ± 0.47 ^b^	72.61 ± 0.44 ^b^	71.50 ± 1.06 ^b^	<0.001
Ash (%)	2.98 ± 0.23	2.91 ± 0.24	2.82 ± 0.26	2.72 ± 0.45	2.72 ± 0.27	2.73 ± 0.19	2.72 ± 0.44	0.417
Protein (%)	15.15 ± 0.32 ^b^	14.55 ± 0.20 ^a^	14.48 ± 0.30 ^a^	14.54 ± 0.53 ^a^	14.46 ± 0.45 ^a^	14.46 ± 0.43 ^a^	14.44 ± 0.64 ^a^	0.012
Fat (%)	21.91 ± 0.60 ^c^	3.94 ± 0.18 ^a^	6.34 ± 0.36 ^b^	3.81 ± 0.37 ^a^	6.05 ± 0.43 ^b^	3.94 ± 0.23 ^a^	6.06 ± 0.82 ^b^	<0.001
Carbs (%)	0.29 ± 0.06 ^a^	6.26 ± 1.18 ^c^	5.34 ± 0.39 ^b^	6.46 ± 0.88 ^c^	5.55 ± 0.30 ^b^	5.97 ± 0.56 ^bc^	5.28 ± 0.54 ^b^	<0.001
pH	6.22 ± 0.01 ^c^	6.16 ± 0.01 ^b^	6.17 ± 0.01 ^b^	6.11 ± 0.03 ^a^	6.16 ± 0.01 ^b^	6.12 ± 0.02 ^a^	6.15 ± 0.01 ^b^	<0.001
WHC (%)	1.07 ± 0.11	1.04 ± 0.01	1.04 ± 0.01	1.04 ± 0.00	1.04 ± 0.01	1.04 ± 0.01	1.04 ± 0.00	0.388
CL (%)	3.20 ± 0.52 ^a^	6.45 ± 0.77 ^c^	5.31 ± 0.58 ^b^	6.69 ± 0.52 ^c^	5.62 ± 0.56 ^b^	6.56 ± 0.61 ^c^	5.48 ± 0.57 ^b^	<0.001
PL (%)	1.36 ± 0.16 ^a^	1.61 ± 0.16 ^b^	1.58 ± 0.16 ^b^	1.59 ± 0.20 ^bb^	1.54 ± 0.23	1.60 ± 0.08 ^b^	1.56 ± 0.16 ^b^	0.043
WL (%)	4.31 ± 0.90 ^a^	12.24 ± 1.36 ^d^	9.48 ± 1.31 ^b^	12.39 ± 1.73 ^d^	10.63 ± 0.57 ^b^	11.16 ± 1.45 c	8.59 ± 0.88 ^b^	<0.001
FL (%)	2.45 ± 0.71 ^a^	5.73 ± 0.31 ^c^	4.49 ± 0.72 ^bc^	3.73 ± 0.69 ^b^	4.14 ± 0.99 ^b^	8.15 ± 2.04 ^d^	6.72 ± 1.80 ^c^	<0.001
TFL (%)	6.76 ± 0.19 ^a^	17.97 ± 1.50 ^d^	13.97 ± 1.62 ^b^	16.12 ± 1.38 ^c^	14.77 ± 1.55 ^b^	19.31 ± 1.00 ^d^	15.31 ± 1.00 ^b^	<0.001

* Different superscript letters (a–d) within the same row indicate significant differences between treatments (*p* < 0.05). WHC = water-holding capacity; CL = cooking loss; PL = purge loss; WL = water loss; FL = fat loss; TFL = total fluid loss.

**Table 2:** Effect of oil type and emulsion ratio on instrumental colour parameters (L*, a*, b*, h*, C*, ΔE) of chicken frankfurters reformulated with inulin-based emulsion gels *.

	Control (C)	Linseed (L)		Walnut (W)		Algal (A)		*p* Value
		1:2:0.5	1:2:1	1:2:0.5	1:2:1	1:2:0.5	1:2:1	
L*	77.26 ± 2.34 ^a^	81.28 ± 1.25 ^b^	82.13 ± 0.98 ^b^	81.39 ± 0.68 ^b^	84.18 ± 1.18 ^c^	82.14 ± 0.33 ^b^	85.70 ± 0.34 ^c^	0.008
a*	6.40 ± 0.21 ^d^	3.54 ± 0.22 ^b^	3.34 ± 0.31 ^b^	3.86 ± 0.09 ^c^	3.29 ± 0.09 ^b^	3.43 ± 0.16 ^b^	2.34 ± 0.03 ^a^	<0.001
b*	12.44 ± 0.24 ^a^	14.92 ± 0.21 ^c^	16.99 ± 0.27 ^d^	13.21 ± 0.21 ^b^	13.68 ± 0.21 ^b^	15.62 ± 0.10 ^c^	16.96 ± 0.08 ^d^	<0.001
h*	62.75 ± 0.87 ^a^	76.64 ± 0.63 ^c^	79.18 ± 1.16 ^c^	73.72 ± 0.14 ^b^	76.48 ± 0.17 ^c^	77.83 ± 0.57 ^c^	82.14 ± 0.12 ^d^	<0.001
C*	13.99 ± 0.24 ^a^	15.34 ± 0.25 ^b^	17.30 ± 0.22 ^c^	13.76 ± 0.23 ^a^	14.07 ± 0.22 ^a^	16.29 ± 0.10 ^b^	17.12 ± 0.08 ^c^	<0.001
ΔE	-	9.37 ± 1.31 ^b^	12.59 ± 1.62 ^c^	7.45 ± 0.81 ^a^	11.28 ± 1.50 ^c^	11.34 ± 0.28 ^c^	17.03 ± 0.31 ^d^	<0.001

* Different superscript letters (a–d) within the same row indicate significant differences between treatments (*p* < 0.05). L* = lightness; a* = redness; b* = yellowness; h* = hue angle; C* = chroma; ΔE = total colour difference relative to the control.

**Table 3:** Effect of oil type and storage time on texture profile analysis (TPA) parameters of chicken frankfurters reformulated with inulin-based emulsion gels *.

O	S	Hardness	Adhesiveness	Springiness	Cohesiveness	Gumminess	Chewiness	Resilience
C (pork fat)	0	2097.39 ± 463.45 ^Ab^	−30.66 ± 10.18 ^b^	0.91 ± 0.01	0.78 ± 0.02 ^b^	1648.02 ± 406.41 ^A^	1503.67 ± 396.20 ^A^	0.42 ± 0.02 ^b^
	30	2203.25 ± 345.62 ^Bb^	−32.79 ± 13.77 ^c^	0.89 ± 0.02	0.76 ± 0.01 ^b^	1692.20 ± 269.08 ^A^	1510.67 ± 253.28 ^A^	0.41 ± 0.01 ^b^
	45	2342.29 ± 355.72 ^B^	−36.67 ± 11.85 ^c^	0.91 ± 0.01	0.76 ± 0.01 ^b^	1799.29 ± 254.36 ^B^	1643.49 ± 230.40 ^B^	0.42 ± 0.01 ^b^
L (1:2:0.5)	0	2194.80 ± 295.66 ^Bc^	−26.67 ± 14.53 ^b^	0.84 ± 0.01	0.72 ± 0.01 ^a^	1590.48 ± 194.18	1345.32 ± 168.44 ^B^	0.36 ± 0.01 ^a^
	30	2007.64 ± 249.89 ^Aa^	−33.17 ± 8.67 ^c^	0.86 ± 0.01	0.73 ± 0.01 ^ab^	1474.61 ± 173.59	1273.95 ± 158.17 ^AB^	0.37 ± 0.01 ^a^
	45	1939.66 ± 337.16 ^A^	−29.52 ± 8.53 ^b^	0.85 ± 0.01	0.74 ± 0.01 ^ab^	1439.65 ± 238.88	1225.28 ± 207.45 ^A^	0.37 ± 0.01 ^a^
L (1:2:1)	0	2011.19 ± 307.08 ^Ab^	−21.62 ± 10.49 ^a^	0.87 ± 0.02	0.73 ± 0.02 ^a^	1474.91 ± 202.57 ^A^	1295.56 ± 187.88 ^A^	0.36 ± 0.01 ^a^
	30	2206.83 ± 402.54 ^Bb^	−24.61 ± 11.21 ^b^	0.86 ± 0.02	0.73 ± 0.01 ^ab^	1621.72 ± 282.66 ^B^	1407.81 ± 266.61 ^B^	0.36 ± 0.01 ^a^
	45	2343.26 ± 284.48 ^B^	−21.16 ± 8.40 ^a^	0.88 ± 0.02	0.75 ± 0.03 ^b^	1756.36 ± 214.07 ^B^	1561.71 ± 212.62 ^C^	0.37 ± 0.02 ^a^
W (1:2:0.5)	0	2092.59 ± 215.58 ^Ab^	−27.68 ± 7.73 ^b^	0.86 ± 0.02	0.70 ± 0.01 ^Aa^	1564.30 ± 149.04 ^A^	1238.74 ± 137.19 ^A^	0.34 ± 0.01 ^Aa^
	30	2303.47 ± 173.74 ^Bb^	−25.08 ± 10.01 ^b^	0.85 ± 0.01	0.71 ± 0.01 ^Aa^	1643.18 ± 112.09 ^A^	1412.28 ± 92.88 ^B^	0.36 ± 0.01 ^Ba^
	45	2416.34 ± 373.21 ^Bc^	−28.94 ± 8.58 ^b^	0.88 ± 0.02	0.75 ± 0.03 B^ab^	1828.75 ± 290.51 ^B^	1605.67 ± 270.98 ^C^	0.39 ± 0.03 ^Cb^
W (1:2:1)	0	2205.37 ± 319.69 ^Ac^	−35.74 ± 11.51 ^c^	0.92 ± 0.27	0.71 ± 0.02 ^a^	1669.37 ± 201.75	1452.70 ± 446.85 ^AB^	0.35 ± 0.01 ^a^
	30	2195.76 ± 267.39 ^Ab^	−38.42 ± 10.79 ^d^	0.86 ± 0.01	0.72 ± 0.01 ^a^	1684.17 ± 171.27	1375.18 ± 138.50 ^A^	0.36 ± 0.01 ^a^
	45	2503.81 ± 182.66 ^B^	−42.78 ± 11.38 ^d^	0.86 ± 0.01	0.71 ± 0.03 ^a^	1795.88 ± 154.23	1557.24 ± 129.97 ^B^	0.35 ± 0.02 ^a^
A (1:2:0.5)	0	1995.39 ± 343.42 ^b^	−29.81 ± 8.32 ^b^	0.88 ± 0.03	0.77 ± 0.03 ^Bb^	1550.40 ± 272.88 ^A^	1367.53 ± 250.82 ^A^	0.39 ± 0.03 ^b^
	30	2185.07 ± 278.93 ^b^	−26.29 ± 8.35 ^b^	0.87 ± 0.01	0.74 ± 0.01 ^Aab^	1636.14 ± 190.52 ^AB^	1435.52 ± 171.75 ^A^	0.37 ± 0.01 ^a^
	45	2263.08 ± 261.04	−25.69 ± 10.04 ^b^	0.88 ± 0.01	0.75 ± 0.01 ^ABab^	1722.12 ± 178.93 ^B^	1695.60 ± 157.85 ^B^	0.38 ± 0.01 ^ab^
A (1:2:1)	0	1819.49 ± 209.97 ^Aa^	−30.61 ± 8.61 ^b^	0.88 ± 0.01	0.77 ± 0.03 ^ABb^	1408.73 ± 183.87 ^A^	1242.80 ± 164.95	0.40 ± 0.03 ^ABb^
	30	1872.24 ± 243.87 ^Aa^	−33.06 ± 9.86 ^c^	0.87 ± 0.01	0.75 ± 0.01 ^Ab^	1525.30 ± 158.42 ^B^	1342.33 ± 147.61	0.38 ± 0.01 ^Aab^
	45	2017.91 ± 212.89 ^B^	−22.96 ± 9.01 ^a^	0.89 ± 0.01	0.80 ± 0.04 ^Bc^	1501.52 ± 210.65 ^B^	1337.76 ± 191.35	0.42 ± 0.02 ^Bb^
O	*p*-value	<0.001	<0.001	0.447	<0.001	0.107	0.120	<0.001
S	*p*-value	<0.001	0.725	0.606	0.007	<0.001	<0.001	<0.001
O × S	*p*-value	0.241	0.172	0.944	0.011	0.275	0.532	0.014

* Different lowercase superscript letters (a–d) within the same column indicate significant differences among oil types (Control, linseed, walnut, algal) at the same storage time (*p* < 0.05). Different uppercase superscript letters (A–C) within the same column indicate significant differences among storage times (0, 30, 45 days) within the same oil type (*p* < 0.05). O = oil type; S = storage time. The O × S term represents the interaction between oil type and storage time. C = Control (pork fat); L = linseed oil; W = walnut oil; A = algal oil.

## Data Availability

The original contributions presented in this study are included in the article. Further inquiries can be directed to the corresponding author.
